# Evaluation of testicular blood flow during testicular torsion surgery in children using the indocyanine green–guided near-infrared fluorescence imaging technique

**DOI:** 10.3389/fped.2023.1272659

**Published:** 2023-10-30

**Authors:** Xiaomeng Liu, Yi Xu, Long Li, Dongsheng Bai

**Affiliations:** ^1^ Department of Urology, Children’s Hospital of Capital Institute of Pediatrics, Beijing, China; ^2^ Graduate School, Chinese Academy of Medical Sciences and Peking Union Medical College, Beijing, China

**Keywords:** testicular torsion, indocyanine green–guided near-infrared fluorescence imaging, imaging time, testicular blood flow, surgical methods

## Abstract

**Objective:**

This study investigates the feasibility of the indocyanine green–guided near-infrared fluorescence (ICG-NIRF) imaging technique in evaluating testicular blood flow during testicular torsion (TT) surgery in pediatric cases.

**Methods:**

We retrospectively analyzed the eight pediatric patients with TT who underwent surgery in our hospital between February and July 2023. The intraoperative two-step method of ICG-NIRF imaging and testicular incision was used to evaluate the testicular blood flow, followed by a selection of different surgical methods. The removed testes were pathologically examined after surgery, and all patients were followed up 1 month after surgery to evaluate testicular blood flow using gray-scale ultrasound and color Doppler flow imaging (CDFI).

**Results:**

Eight pediatric TT patients aged 1–16 years, with a median age of 11.5 years, were enrolled. Time from the onset ranged from 4 to 72 h (mean 26.13 ± 25.09 h). A total of eight testes were twisted, including four on the left side and four on the right side. The twisting direction of the testes was clockwise in four cases and counterclockwise in four cases. The rotation of torsion was 180°–1,080° (mean 472.5° ± 396°). There was no statistically significant difference in the imaging time between the four patients with testicular blood vessel imaging on both the torsional and normal sides (*P* > 0.05). The postoperative recovery was uneventful, with no complications during the follow-up period of 1 month. The postoperative histopathological results of three patients who underwent orchiectomy showed extensive hemorrhage, degeneration, and necrosis of the testicular tissue. Among the five patients who underwent orchiopexy, a gray-scale ultrasound and CDFI examinations showed uniform internal echo of the testes and normal blood flow signals in four patients. One patient with no testicular blood vessel imaging on the torsional side showed uneven internal echo of the testis and no blood flow signals.

**Conclusion:**

ICG-NIRF imaging is a feasible method to evaluate testicular blood flow during TT surgery. Testicular blood vessel imaging within 5 minutes after ICG injection might be the basis for testicular retention during TT surgery.

## Introduction

Testicular torsion (TT) is a urological emergency. Most scholars agree that the “golden time” for testes survival is within 6 hours from the onset of TT. The earlier the time of treatment, the greater the chance of saving the testes. With the prolongation of the onset time, the possibility of retaining the testes gradually decreases (1, 2). It has been demonstrated that the average testicular resection rate during TT surgery is 39% (20%–60%), and the long-term testicular loss rate is 49%, suggesting a high rate of long-term testicular atrophy (2). Further studies have estimated that the overall rate of testicular loss in children and adolescents approaches 60% (2).

The selection of surgical methods in TT surgery is mainly based on observing the testicular blood flow after detorsion. At present, testicular blood flow is assessed by intraoperative observation of testicular color (3, 4). It can also be evaluated by observing hemorrhage after incising the testicular parenchyma (3, 5, 6). However, regardless of which method is used, it is largely affected by the subjective factors of surgeons and lacks reliable objective evidence. Until now, there is still no objective criterion for testicular retention during TT surgery.

Indocyanine green (ICG) is a three-carbon cyanine dye that can emit fluorescence when irradiated by near-infrared (NIR) at a specific wavelength and can be received and converted to imaging by a specific device (3, 4). An ICG-guided NIR fluorescence (ICG-NIRF) imaging technique has been widely used in adult (7–10) and pediatric (11–14) surgery, including angiography, cholangiography, localization of sentinel lymph nodes, and evaluation of perfusion of various tissues, organs, or tumors.

This study aims to report the application of the ICG-NIRF imaging technique in assessing testicular blood flow during TT surgery in Children's Hospital of Capital Institute of Pediatrics and to explore the feasibility of the ICG-NIRF imaging technique in evaluating testicular blood flow during TT surgery in pediatric cases.

## Materials and methods

### Clinical data

This study retrospectively analyzed pediatric patients with evaluated testicular blood flow using the ICG-NIRF imaging technique during TT surgery in our center from February to July 2023. Eight patients with TT (two with cryptorchidism) aged 1–16 years, with a median age of 11.5 years, were enrolled. Time from the onset ranged from 4 to 72 h (mean 26.13 ± 25.09 h). A total of eight testes, including four on the left side and four on the right side, were torsional. The twisting direction of the testes was clockwise in four cases and counterclockwise in four cases. The rotation of torsion was 180°–1,080° (mean 472.5° ± 396°) (Table 1).

**Table 1:** Clinical data and surgical steps and postoperative follow-up of TT patients managed with ICG-NIRF imaging.

Case no.	Age (years)	Time from the onset (h)	Torsional side	Direction and rotation of torsion	ICG-NIRF imaging in the blood vessels of bilateral testes and imaging time (s)		Hemorrhage in the testicular parenchyma after incision	Surgical methods	Postoperative findings of torsional testes	
					Torsional side	Normal side			Ultrasonography	Histopathology
1	16	72 h	Left	Counterclockwise 360°	N/A	30	No	Orchiectomy	N/A	Extensive hemorrhage, degeneration, and necrosis
2	11	9 h	Right	Clockwise 180°	44	35	N/A	Orchiopexy	Uniform internal echo, normal blood flow signals	N/A
3	13	4 h	Right	Clockwise 180°	25	25	N/A	Orchiopexy	Uniform internal echo, normal blood flow signals	N/A
4	1 year 10 months	48 h	Left (cryptorchidism)	Counterclockwise 540°	N/A	30	Yes	Orchiopexy	Uneven internal echo, no blood flow signals	N/A
5	12	10 h	Left	Counterclockwise 180°	N/A	32	No	Orchiectomy	N/A	Extensive hemorrhage, degeneration, and necrosis
6	2 years 3 months	42 h	Left (cryptorchidism)	Counterclockwise 1,080°	N/A	40	No	Orchiectomy	N/A	Extensive hemorrhage, degeneration, and necrosis
7	11	20 h	Right	Clockwise 1,080°	35	30	N/A	Orchiopexy	Uniform internal echo, normal blood flow signals	N/A
8	12	4 h	Right	Clockwise 180°	20	20	N/A	Orchiopexy	Uniform internal echo, normal blood flow signals	N/A

### Surgical steps and postoperative follow-up

**Figure 1:**
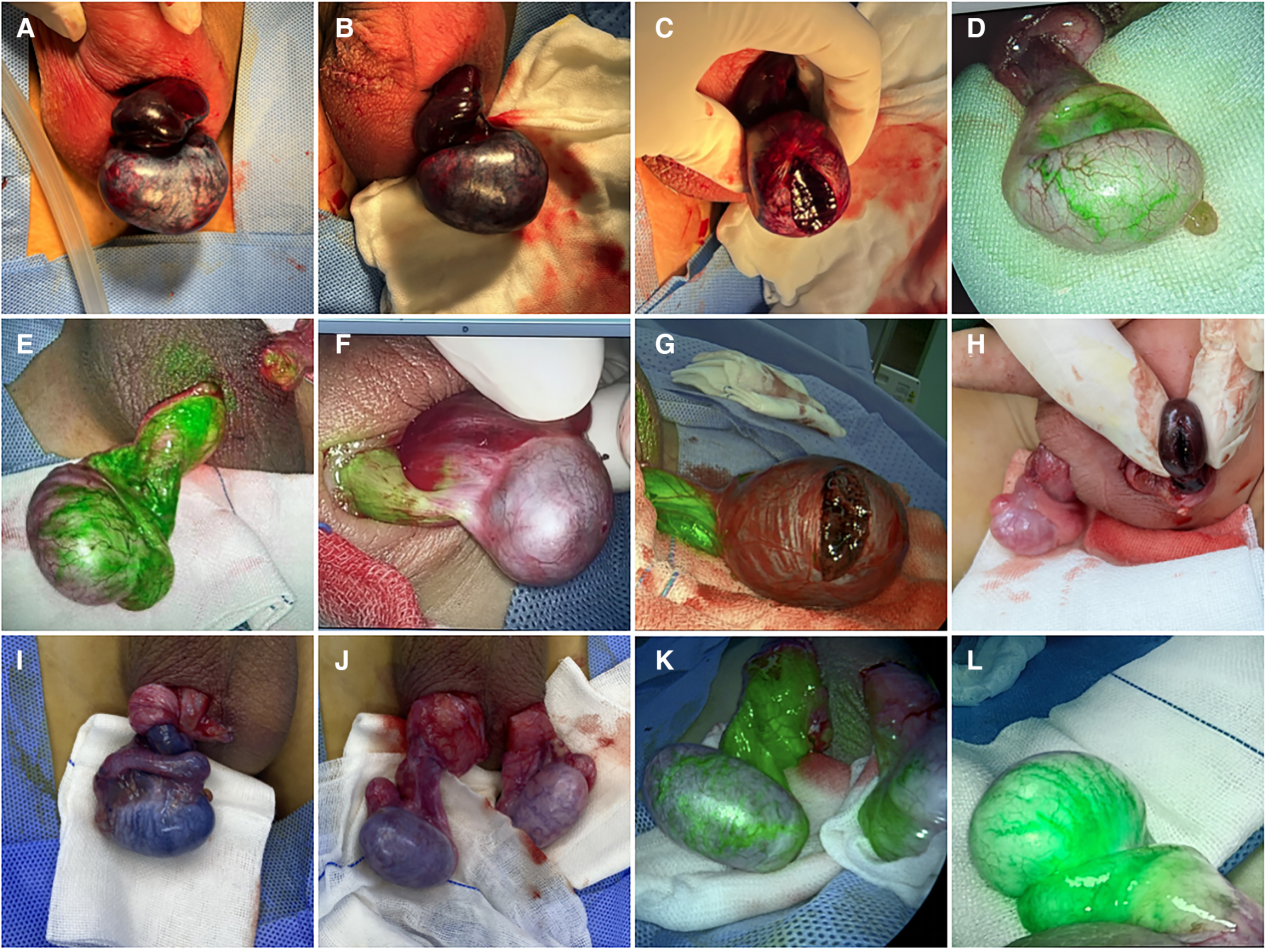
Intraoperative TT images of eight cases. (**A**) After detorsion; (**B**) after applying warm saline-soaked gauze to the torsional testis for 10 min; (**C**) testicular parenchyma was incised when the fluorescence was not observed within 5 min in case 1. Torsional testis blood vessel imaging was observed in cases 2 (**D**) and 3 (**E**) and was not observed within 5 min in case 4 (**F**). Testicular parenchyma was incised when the fluorescence was not observed within 5 min in cases 5 (**G**) and 6 (**H**). (**I**) Before detorsion; (**J**) after applying warm saline-soaked gauze to torsional testis after detorsion for 10 min; (**K**) torsional testis blood vessel imaging was observed in case 7. (**L**) Torsional testis blood vessel imaging was observed in case 8.

The patients were in the supine position after general anesthesia. A transverse incision was performed on the torsional side of the central scrotum to expose the torsional testis. Then, a warm saline-soaked gauze was applied to the testis for 10 min after detorsion. After intravenous injection of ICG (dosage 0.3 mg/kg) (15), the bilateral testicular blood vessel imaging time was recorded. If the testicular blood flow on the torsional side was observed within 5 min, bilateral orchiopexy was performed. If the fluorescence could not be confirmed within 5 min, the testes were incised along the longitudinal axis to the testicular parenchyma to observe the hemorrhage of the testicular tissue further. If there was testicular parenchymal hemorrhage, the tunica albuginea of the testis was sutured, and bilateral orchiopexy was performed. If there was no hemorrhage in the testicular parenchyma, an orchiectomy was performed, and the contralateral testis was fixed (Figure 1). One month after the surgery, all patients underwent a gray-scale ultrasound and color Doppler flow imaging (CDFI) to evaluate the morphological structure and hemodynamic changes of the testis. Histopathological examinations were performed on the removed testes of the patients undergoing orchiectomy (Table 1).

### Statistical analysis

The data were presented as the mean ± standard deviations, calculated using GraphPad Prism version 9.0. The Shapiro–Wilk test was performed to evaluate the data distribution. Due to the normal distribution of the data, a paired *t*-test was performed for further analysis. A *P*-value of <0.05 was considered statistically significant.

## Results

**Figure 2:**
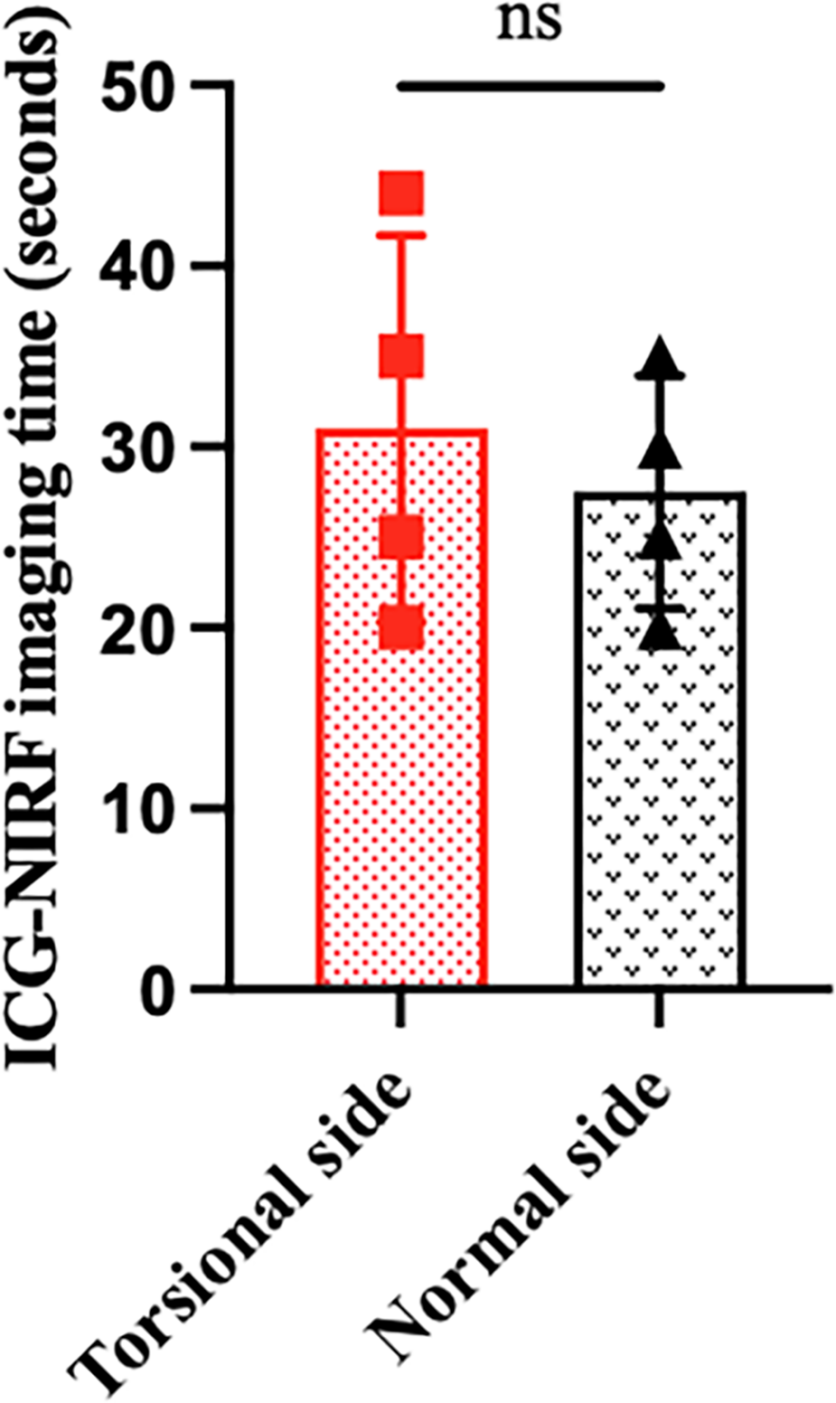
ICG-NIRF imaging time of testicular blood vessels between the torsional and normal sides.

**Figure 3:**
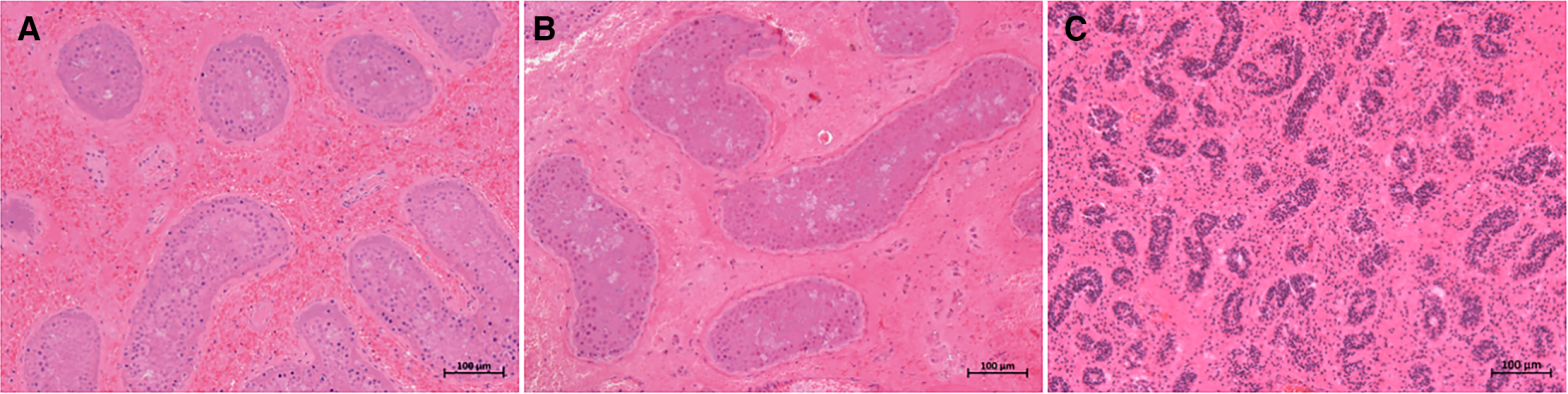
Histopathological results of excised testes in three cases. (**A–C**) presented testicular extensive hemorrhage, degeneration, and necrosis in cases 1, 5, and 6, respectively. Staining method: hematoxylin–eosin (H&E) staining; magnification ×100.

**Figure 4:**
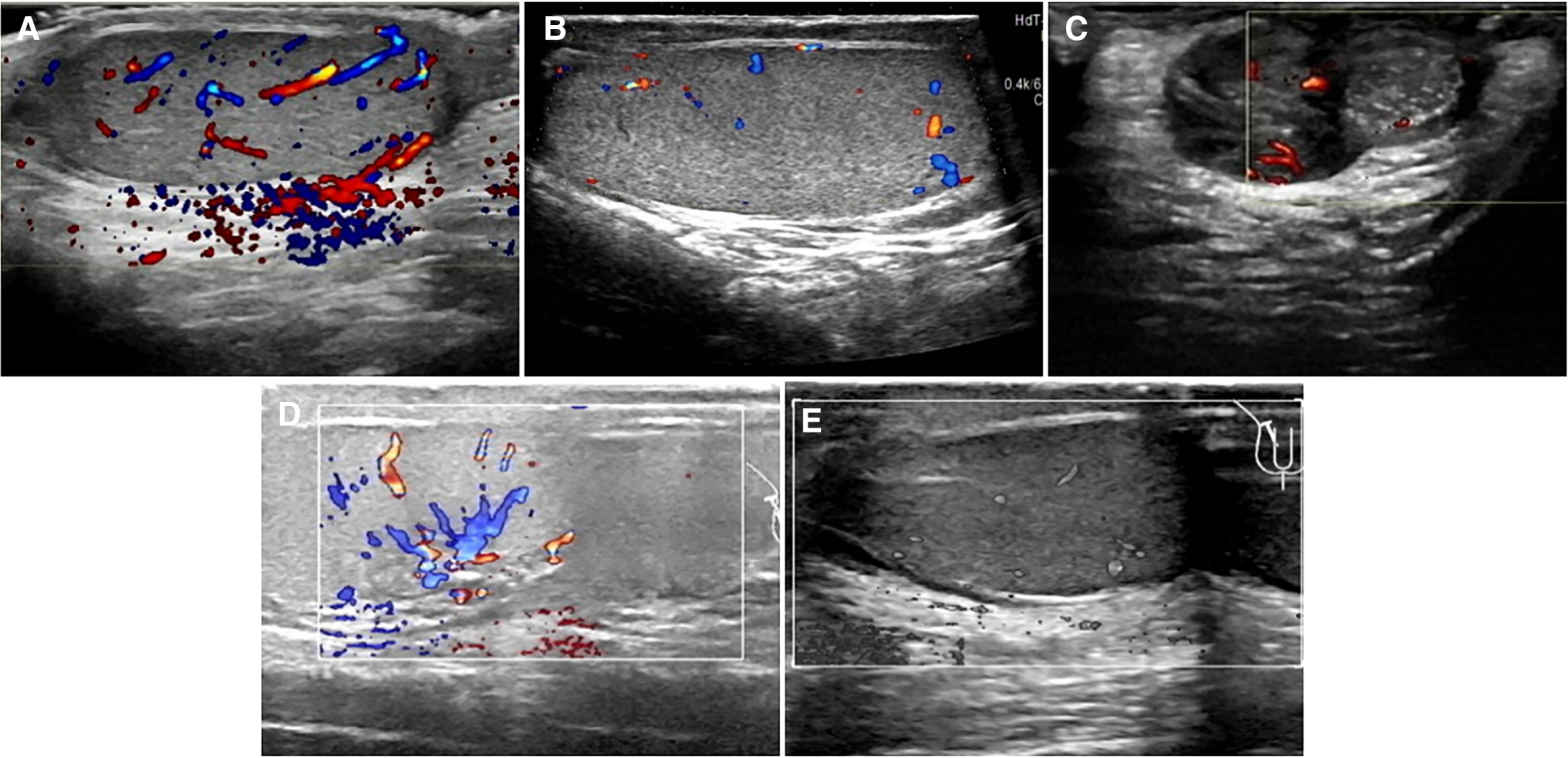
Postoperative ultrasonographic findings of torsional testes in five cases. (**A**) Uniform internal echo in the testis; CDFI: normal blood flow signals in the testis, stellate blood flow signals in the tunica albuginea in case 2. (**B**) Uniform internal echo in the testis; CDFI: normal blood flow signals in the testis, stellate blood flow signals in the tunica albuginea in case 3. (**C**) Testis presented uneven internal echo with slightly punctate stronger echo; CDFI: several blood flow signals around the diseased testis but no obvious signals within it in case 4. (**D**) Testicular parenchyma echo was uniform; normal echo was observed around the testis; CDFI: normal blood flow signals were found in the testis in case 7. (**E**) Internal echo of the testis was uniform; no cystic or solid foci were observed; CDFI: normal blood flow signals in the testis in case 8.

All eight patients in our center were diagnosed with TT by a preoperative ultrasonography scan, confirmed by surgical exploration. All the surgeries were successfully completed. The intraoperative ICG-NIRF imaging technique was used to evaluate the testicular blood flow. Five patients underwent orchiopexy, and three patients underwent orchiectomy. Among them, testicular blood vessel imaging was observed 20–44 s after ICG injection in four patients, and orchiopexy was performed. In the remaining four patients, no testicular blood vessel imaging was observed within 5 min after ICG injection. These patients underwent testicular incision to observe testicular parenchymal hemorrhage. Orchiopexy was performed in one case because of testicular parenchymal hemorrhage without ICG leakage. The other three patients had no hemorrhage and underwent orchiectomy (Figure 1, Table 1). In all eight patients, the normal side of the testes showed blood vessel imaging between 20 and 40 s after ICG injection. There was no statistically significant difference in the imaging time among the four patients with testicular blood vessel imaging on both the torsional and normal sides (*P* > 0.05) (Figure 2). All patients recovered well after surgery, with no complications related to surgery or ICG use. The postoperative histopathological results of the three patients who underwent orchiectomy showed extensive hemorrhage, degeneration, and necrosis of the testicular tissue (Figure 3). Among the five patients who underwent orchiopexy, a gray-scale ultrasound and CDFI examination at 1 month after surgery showed a uniform internal echo of the testes and normal blood flow signals in four patients. One patient with no testicular blood vessel imaging on the torsional side showed uneven internal echo of the testis with no blood flow signals by ultrasound and CDFI 1 month after surgery (Figure 4).

## Discussion

As an organ with both endocrine and reproductive functions, the testis is of great importance to men. Jacobsen et al. reviewed articles examining changes in testicular function after TT and stated that there is no evidence that it affects sex hormone levels including luteinizing hormone (LH), follicle-stimulating hormone (FSH), and testosterone. However, it can affect semen quality. These patients experience decreased sperm motility and reduced total sperm count, possibly leading to reduced fertility. Studies in animals and humans have also confirmed the presence of damage to the contralateral testis after TT, which may result from multiple factors (16). Other studies have indicated that orchiectomy might negatively affect patients’ mental health, such as anxiety and shame (17).

Inappropriately selecting surgical methods has a great impact on the future life of patients. It is particularly important to choose the right intraoperative surgical methods. In terms of endocrine functions, Arap et al. compared the effects of different surgical methods on the endocrine function of patients with TT, and they found that the average FSH level in blood samples in the orchiectomy group is obviously higher than that in the orchiopexy group. There is no statistical difference in serum LH and testosterone levels between the two groups (18). Similarly, in prepubertal patients with TT, Osemlak et al. indicated that serum FSH levels are higher in the orchiectomy group than in the orchiopexy group. However, they did not compare the FSH levels in those pubertal patients with TT treated with two different surgical methods (19). In terms of reproduction, a study in rats has shown that orchiopexy or orchiectomy has no effect on the future fertility in TT with a twisting time greater than 4 h (20). In addition, in patients with TT, Arap et al. revealed that the sperm activity and morphology in the orchiectomy group are significantly better than those in the orchiopexy group. They speculated that it might be related to the release of abnormal sperms from the retained testes or the anti-sperm antibodies produced after the blood–testis barrier is damaged. At the same time, they also suggested that the production of the anti-sperm antibody is independent of the choice of surgical methods (18). However, in the study of Zhang et al., they reported that the younger the age of TT onset, the smaller the negative impact on the first pregnancy rate and time to pregnancy. Moreover, the first pregnancy rate of the orchiectomy group is lower than that of the orchiopexy group, and the time to pregnancy is much longer (21).

There is still some controversy about whether to perform contralateral orchiopexy during TT surgery. The guidelines for pediatric urology of the European Association of Urology have pointed out that the contralateral orchiopexy should be performed in children with TT. However, they did not mention the reason (22). Studies have shown that approximately 60%–80% of children born with testicular bell clapper deformity also have this deformity in the contralateral testis, which makes it possible for the contralateral testis to develop a new torsion. Therefore, contralateral orchiopexy is recommended (16). Moore et al. reviewed a large number of articles on the use of orchiopexy during TT surgery and suggested that contralateral orchiopexy may prevent torsion of the contralateral testis in the future. They also demonstrated that a few scholars do not support contralateral orchiopexy because they believe that the risk of contralateral TT is very low and that this risk is lower than the risk of surgical complications (23).

In 1959, the US Food and Drug Administration approved ICG for clinical use (7, 12, 24). It can be injected directly into the blood, and most (98%) of the ICG in the blood binds to plasma proteins and excreted in a free form through bile via the hepatobiliary pathway (12, 25). No allergy or other adverse reactions to ICG have been reported in the literature. Since ICG contains sodium iodide, the only contraindication for its use could be in people allergic to iodine (26). None of our eight patients experienced allergic reactions or other ICG-related complications after intravenous injection of ICG. Therefore, we believe ICG might be safe for testicular blood flow imaging during TT surgery.

At present, the feasibility of applying the ICG-NIRF imaging technique to evaluate testicular blood flow during TT surgery remains unclear, and only three case reports have been published (4, 15, 27). Among the five reported TT cases, testicular blood vessel imaging was observed after intravenous ICG injection, four of which took less than 1 min (30–45 s) and one did not report the imaging time and finally underwent orchiectomy to retain the testis. Negative cases of testis blood vessel imaging have not been reported. It has also been reported that the testis should be observed for 10 min after incision, a warm sponge should be applied around the testis to help blood flow recovery, and hemorrhage of testicular parenchyma could be used as an evaluation criterion for the selection of surgical methods during TT surgery (6). At present, there is no standard to be used for the ICG-NIRF imaging technique in TT surgery. In this study, we determined that 5 min is sufficient to assess the recovery of testicular blood flow after ICG injection.

In the present study, four cases undergoing orchiopexy showed bilateral testicular blood vessel imaging within 1 min after ICG injection, similar to those reported above. In the other four cases, no blood vessel imaging was observed in the torsional testes within 5 min after ICG injection. Therefore, the testes were incised to observe whether the testicular parenchyma hemorrhaged. Orchiectomy was performed in three cases without hemorrhage, and orchiopexy was performed in one patient with testicular parenchymal hemorrhage. Four cases with testicular blood vessel imaging on the torsional sides showed uniform internal echo and normal blood flow signals during the 1-month follow-up. The histopathological results of three cases who underwent orchiectomy showed testicular necrosis, consistent with the intraoperative observation. An ultrasound follow-up of the other patient at 1 month after surgery showed uneven internal echo and no blood flow signals in the torsional testis. We consider that this result might be because hemorrhage after testicular incision originates from venous congestion or hemorrhage in the tunica albuginea blood vessels. Arda et al. reported that venous and arterial hemorrhage should be distinguished when performing a testicular incision to observe hemorrhage and that tunica albuginea hemorrhage could not be used as a true indicator of testicular activity (6). However, data for more cases are needed to support this opinion.

There was no statistical difference in the time of testicular blood vessel imaging between the torsional sides and the normal sides, suggesting that there was no significant difference in the testicular blood flow between the torsional testis after detorsion and warm compress and the normal testis, which may indicate that the good blood flow of the torsional testis and the testis could be retained. The postoperative follow-up ultrasound in four cases also confirmed this finding.

**Figure 5:**
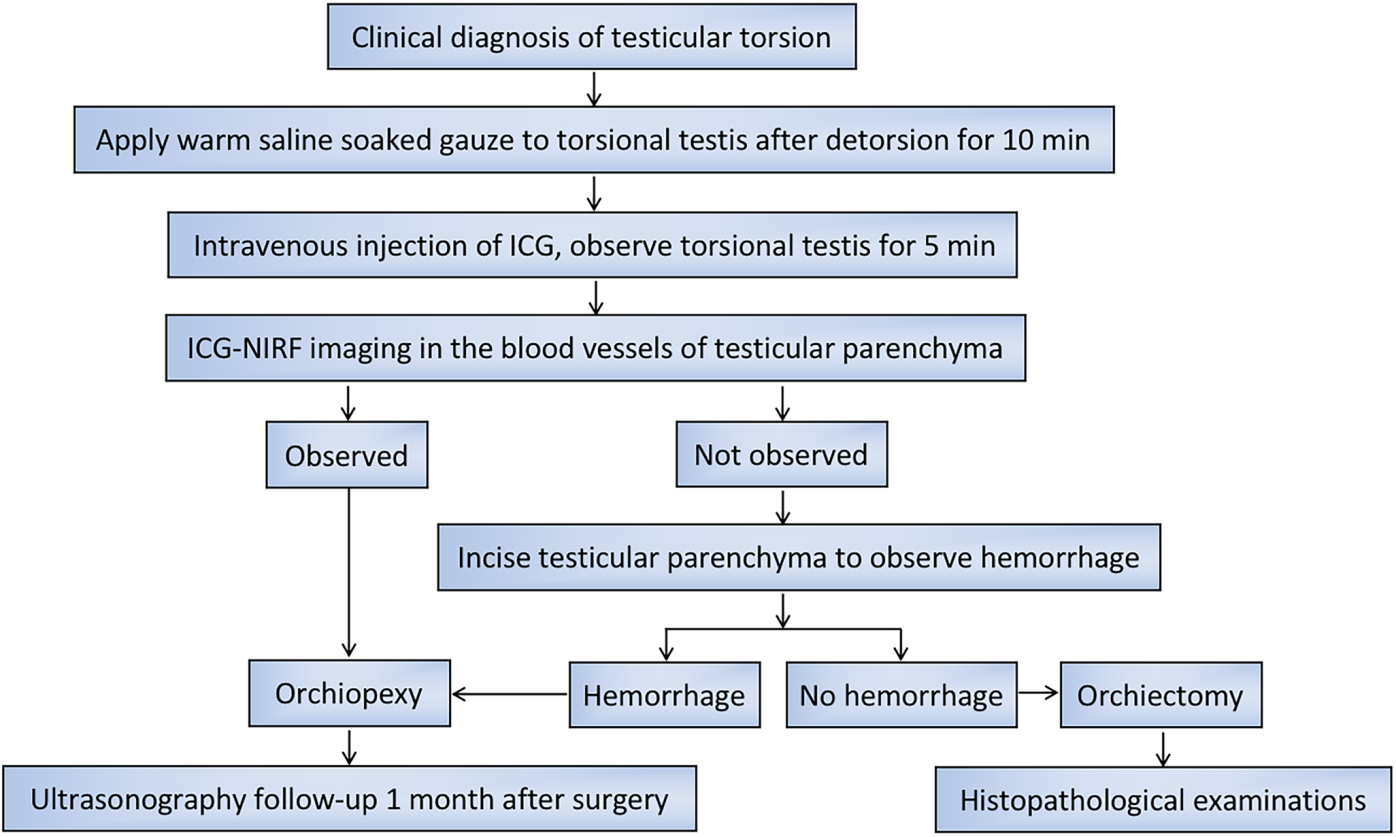
Flow chart for the management of operation and postoperative follow-up in pediatric patients with TT.

In summary, after testicular detorsion, we recommend that a warm saline-soaked gauze could be applied to the testis for 10 min, followed by intravenous ICG injection. The testes showing blood vessel imaging within 5 min after ICG injection should be retained, and those that are not imaged within 5 min should be removed (Figure 5).

### Strengths and limitations

This is the first study to evaluate testicular blood flow during TT surgery using the ICG imaging time. This study suggests that the ICG-NIRF imaging technique could provide an objective basis for evaluating testicular blood flow during TT surgery. However, it has several limitations. First, the small sample size of this study and experience from a single center may lead to biases in interpreting the results. Moreover, the postoperative follow-up time is relatively short, and the long-term evaluation of the torsional testes function is lacking. Therefore, we will further increase the number of study cases and carry out a long-term follow-up.

## Conclusion

The ICG-NIRF imaging technique is safe and easy to operate. We point out that the ICG-NIRF imaging technique is feasible for evaluating testicular blood flow during TT surgery. The application of this technique may help avoid the removal of functional testes and the retention of necrotic testes during TT surgery. The torsional testis with blood vessel imaging within 5 min after ICG injection might be the basis for testicular retention during TT surgery. Those that are not imaged within 5 min might be the basis for testicular removal.

## Data Availability

The original contributions presented in the study are included in the article/Supplementary Material, further inquiries can be directed to the corresponding author.
